# Topic prediction for tobacco control based on COP9 tweets using machine learning techniques

**DOI:** 10.1371/journal.pone.0298298

**Published:** 2024-02-15

**Authors:** Sherif Elmitwalli, John Mehegan, Georgie Wellock, Allen Gallagher, Anna Gilmore

**Affiliations:** Tobacco Control Research Group, Department for Health, University of Bath, Bath, United Kingdom; Roma Tre University: Universita degli Studi Roma Tre, ITALY

## Abstract

The prediction of tweets associated with specific topics offers the potential to automatically focus on and understand online discussions surrounding these issues. This paper introduces a comprehensive approach that centers on the topic of "harm reduction" within the broader context of tobacco control. The study leveraged tweets from the period surrounding the ninth Conference of the Parties to review the Framework Convention on Tobacco Control (COP9) as a case study to pilot this approach. By using Latent Dirichlet Allocation (LDA)-based topic modeling, the study successfully categorized tweets related to harm reduction. Subsequently, various machine learning techniques were employed to predict these topics, achieving a prediction accuracy of 91.87% using the Random Forest algorithm. Additionally, the study explored correlations between retweets and sentiment scores. It also conducted a toxicity analysis to understand the extent to which online conversations lacked neutrality. Understanding the topics, sentiment, and toxicity of Twitter data is crucial for identifying public opinion and its formation. By specifically focusing on the topic of “harm reduction” in tweets related to COP9, the findings offer valuable insights into online discussions surrounding tobacco control. This understanding can aid policymakers in effectively informing the public and garnering public support, ultimately contributing to the successful implementation of tobacco control policies.

## Introduction

Due to the influence and reach of content on social media platforms, it is imperative to explore Twitter data when investigating topics that are part of ongoing public policy debates and processes. Tweets can shed light on unfiltered opinions and sentiments, spread of misinformation, industry influence and potential problems policy makers may face due to public backlash. Furthermore, while social media has many benefits in connecting people and communities, it also has a lot of drawbacks. Social media debate can readily devolve into arguments which can include content considered toxic, biased, or can be delivered using insensitive language [1], all of which can shape public perceptions and thus impact on policy development and progression. A particularly relevant and topical case study for exploring these issues further is e-cigarette regulation.

There is a wide literature on topic modelling techniques and their application. Vayanski & Kumar offer an extensive review of topic modeling methods, tracing their historical trajectory, and present an array of applications across text classification, sentiment analysis, and document clustering [2]. More specifically relevant to this paper, Jelodar et al. provide a comprehensive survey of Latent Dirichlet Allocation (LDA) based topic modeling approaches, delving into research developments, contemporary trends, and the intellectual framework underpinning topic modeling [3], with Aziz et al. giving an example of these methods applied in the field of finance [4].

Topic modelling techniques have previously been used in the tobacco domain. Shah et al. combined topic modeling with qualitative content analysis to detect and elucidate sales offers of electronic nicotine delivery systems (ENDS) and related products on Instagram, showcasing the utility of these methods in comprehending product promotion and sales on social media platforms [5].

There is much debate around e-cigarettes, including their potential health impacts, their potential role in reducing tobacco consumption, and the involvement of transnational tobacco companies in such products, all of which has contributed to a large amount of interest from both policymakers and the public in their regulation [6–8]. For instance, the 9^th^ Conference of the Parties (COP9) to the World Health Organization (WHO) Framework Convention on Tobacco Control (FCTC)—an international treaty which provides governments with guidelines for reducing the demand and supply of tobacco products [9], included discussion around e-cigarette regulation [10].

E-cigarette regulation is often discussed within the context of tobacco ‘harm reduction’, whereby the debate centers around the extent to which such products are an effective tool for reducing the harms of tobacco products and one’s perspective on this is likely to influence their views on how such products should then be regulated [11]. In recent years, use of the term ‘harm reduction’ within the context of tobacco control has been growing, including by transnational tobacco companies that have expanded their product portfolios to include products which don’t contain tobacco [12].

News and opinions regarding e-cigarette regulations are widely disseminated on social media platforms, which can have a significant impact on public perceptions of the use of such products [13]. One study investigated vaping-related tweets featuring an image promoting vaping uploaded and retweeted by Australian users to see how e-cigarettes were depicted and marketed on Twitter, and how this evolved and trended over time. Some Twitter users appeared to be making a concentrated effort to advertise e-cigarettes as health-promoting and smoking cessation tools to oppose Australian regulations and lobby for a more permissive attitude to personal vaporizers, prompting the need for further study to understand the online behavior of vaping advocates, and to identify whether this is part of a larger coordinated effort to undermine regulation of tobacco related products [14].

Lazard et al. employed a text mining technique to explore replies to the Food and Drug Administration’s (FDA) e-cigarette restrictions on Twitter. A total of nine topics were identified, including first reactions to the FDA’s e-cigarette restrictions, whether the restrictions would help or hurt public health, and how the restrictions would affect the developing e-cigarette sector. The study found that negative or mixed reactions dominated the topics, suggesting that based on the shared information, Twitter users were not in favor of the e-cigarette restrictions [15].

In a similar vein, Myslín et al. created a supervised prediction model to identify e-cigarette supporters on Twitter and assess their online behavior regarding common topics, using textual elements from recent tweet content and tweeter profile biography data to develop predictive models. It was discovered that 10% of the user dataset were proponents of e-cigarettes, and those users tweeted two to five times more often than the 90% of Twitter users in the dataset that were not proponents [16].

Industry involvement is one factor why social media may appear to be less supportive of e-cigarette regulation than traditional tobacco regulation. Robertson et al. conducted a study to examine Twitter data to learn more about tobacco industry strategies, arguments, and allies, and to establish whether positive e-cigarette messaging on Twitter had links to the tobacco industry. The study investigated Twitter data surrounding the WHO FCTC Eighth Session of the Conference of the Parties (COP8), with 9089 tweets collected October 1–9, 2018, containing the hashtag #COP8FCTC. The study used manual coding and machine learning to classify the content and sentiment of the tweets. It employed a method of investigation based on publicly accessible data to classify the most active Twitter users and investigate tobacco industry ties. To show interactions and discover communities, network analysis was employed. “Next-generation Products” (NGP) supporters with links to organizations funded directly and indirectly by Philip Morris International (PMI) made up most of the most active tweeters, which was consistent with aims in PMI’s 2014 corporate affairs strategy to “amplify and leverage the debate on harm reduction” [17].

However, some public support for tobacco control policy has been previously identified on Twitter. In a study which looked at how the public reacted to the UK Government’s efforts to implement policy on standardized tobacco packaging on Twitter, in general, more Tweets were observed in support of the policy (49%) than against it (19%) [18]. Support for the policy, Twitter user’s geographical location and association, and evidence citation and quality were all investigated using content analysis.

The aim of this paper is to use Twitter discussion around e-cigarette regulation (utilizing tweets collected during COP9) as a case study to build upon previous research using machine learning for topic modelling and sentiment analysis. By doing this, we aim to identify the usefulness of such approaches for gaining further understanding of ongoing policy debates. We show how topic modelling can isolate tweets discussing themes/language relevant to tobacco control (for example “harm reduction”) and demonstrate how the sentiment and toxicity of such tweets can be analyzed, thus helping to inform policymakers on public perceptions of issues related to the approach of public health and tobacco control advocates.

## Methods

Digital Methods Initiative Twitter Capture and Analysis Toolkit (DMI-TCAT) was used to collect tweets relevant to COP9. DMI-TCAT enables researchers to collect tweets on an ongoing basis through Twitter’s Streaming API which gives a representative sample of tweets proportional to the total volume of tweets being posted at any one time [19]. Between 07/11/2021 and 22/11/2021 tweets were collected containing the hashtags #COP9 and #COP9FCTC resulting in a dataset of 7376 tweets in several languages along with metadata such as the user ID and the retweet status. To ensure a coherent English analysis, the Google Translate API was used for tweets translation, given its high performance relative to other techniques [20].

The tweets were preprocessed (as described below) to ensure that the tweet text was suitable for topic modelling. The different topics present in the tweets’ corpus were then identified using topic modelling. Finally, only tweets for one of the topics which we identified as most relevant to the e-cigarette regulatory debate, and which we labelled “harm reduction”, were included for further processing, including analysis of sentiment and toxicity. The correlation between tweets’ sentiment scores and their associated number of retweets were calculated to find whether there is a direct relation between the reach of the tweet and the sentiment of the message. Python was chosen for implementing the utilized algorithms due to its versatility and robust ecosystem for natural language processing tasks.

### A) Datasets

In the context of this research, a subset comprising 1000 tweets from the COP9 dataset underwent manual annotation to determine their associated sentiment. Sentiments were categorized into three classes: -1 for negative sentiment, 0 for neutral sentiment, and 1 for positive sentiment. This manual annotation process serves as the foundation for fine-tuning the sentiment analysis model.

Simultaneously, the sentiment analysis model was initially trained using the IMDB dataset. The IMDB dataset is a well-established resource in natural language processing (NLP) research and machine learning applications, particularly in the domain of sentiment analysis. It consists of 50,000 movie reviews, all composed in English and labeled as either "positive" or "negative" [21]. This dataset provides the initial framework for the sentiment analysis model, which subsequently underwent fine-tuning using the manually annotated COP9 dataset.

### B) Tweets preprocessing and cleaning

Text cleaning and pre-processing involves reducing the tweets to individual words that can be used in the machine learning predictive models for sentiment analysis and topic modelling. This involves several steps. First, tweet text is split into individual words, converted into either lower or upper case and punctuation is removed to make the dataset consistent [22]. Second, words like “the”, “and”, “before”, “while”, and others that do not contribute to the context or content of the tweet are removed from the dataset [23]. Third, both stemming, and lemmatization can be applied with the purpose of reducing a word’s inflectional and occasionally adequate related forms to a single base form [24].

In this study, the preprocessing pipeline for tweets involved a series of steps to improve text quality and uniformity. Initially, the entire tweet content was converted to lowercase for consistency. Subsequently, hashtags, emoticons, hyperlinks, and non-letter characters were removed, retaining only alphanumeric content. Standalone numbers and punctuation marks were also eliminated to focus on textual elements. The processed text underwent tokenization and stopword removal to maintain meaningful information. Finally, the tweet dataset experienced spaCy’s lemmatization to reduce words to their base form, relying on spaCy’s statistical models and parts-of-speech information. Lemmatization is valuable for preserving the sentiment of the original words, which is crucial for accurate sentiment classification, and for maintaining the semantic meaning of words—an essential aspect for identifying and distinguishing topics [25]. However, stemming was deliberately excluded from the preprocessing pipeline, prioritizing accuracy over computational efficiency.

### C) Topic modelling

**Fig 1:**
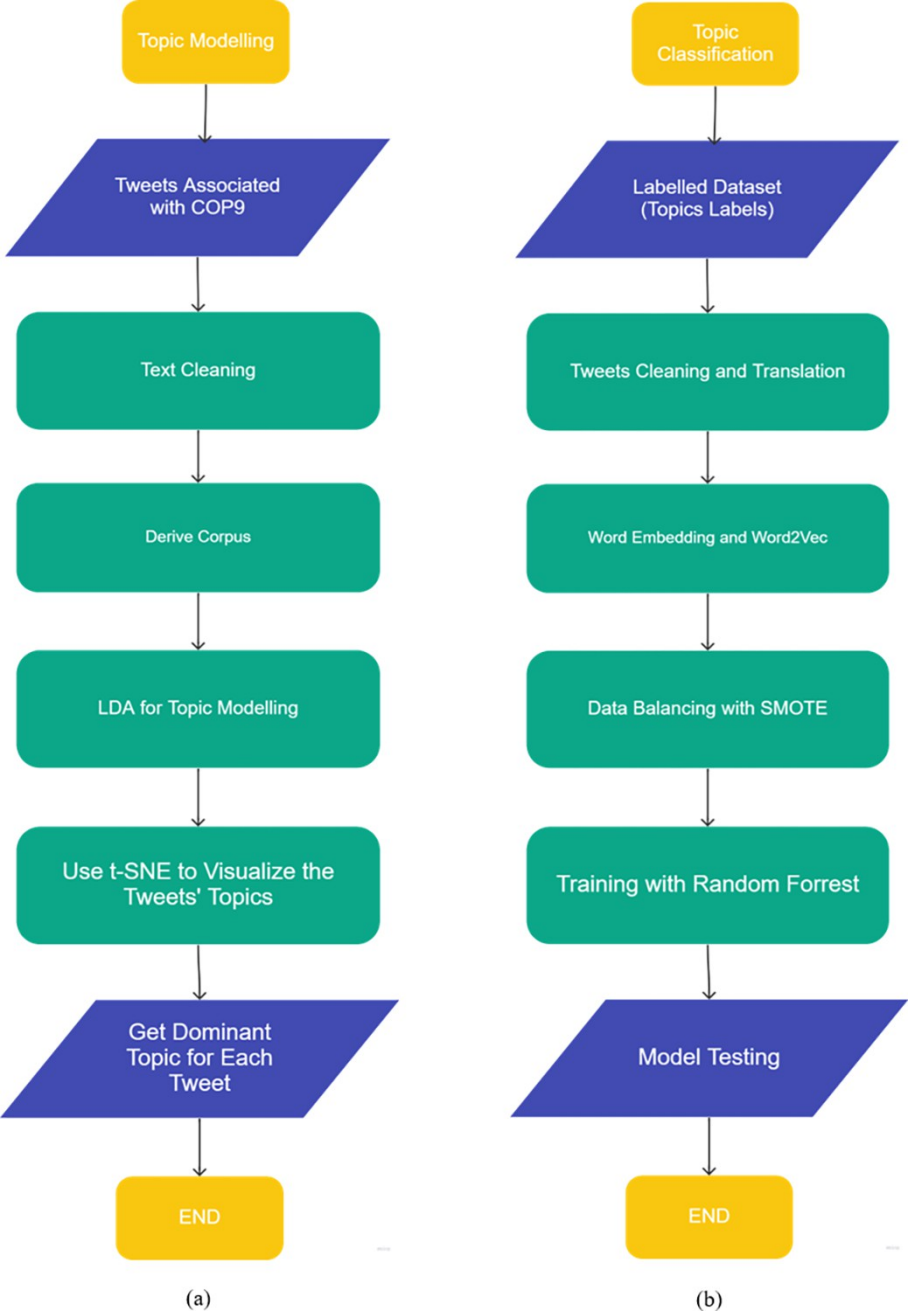
The flowchart of (a) Topic modelling, and (b) Topic classification.

Topic modelling is an unsupervised technique of classifying or extracting themes by finding patterns, in a similar way to how clustering algorithms divide data into sections based on a commonality. Using topic modelling we can learn about the different topics present in the text, by extracting the patterns of word clusters and term frequency, the dataset can be divided into clusters based on these topics as shown in Fig 1A [26].

Two methods were employed in this research for preparing inputs to the topic modelling and sentiment analysis algorithms, word embedding and TF-IDF. We used word2vec for word embedding, which includes models like skip-gram and continuous bag-of-words (CBOW). Skip-gram allows us to start with a word and guess the words that will most likely follow it. On the other hand, Continuous bag-of-words allows for the prediction of a word that is likely to appear in a group of words [27].

#### 1. Latent Dirichlet allocation

Latent Dirichlet allocation (LDA) is an unsupervised Bayesian statistical model that identifies the co-occurrence of terms in a textual corpus and exploits this information to reveal the corpus’s latent topical structure. The structure is made up of a number of topical groupings or subjects, each of which is defined by a set of keywords specific to that topic. This is due to LDA’s two basic assumptions: (1) every document in a corpus is made up of a limited number of topics, and (2) every word in a document may be allocated to one or more of the topics [28].

#### 2. Data balancing

The number of linked tweets might vary greatly depending on the number of subjects. As a result, the final dataset is imbalanced. Resampling approaches, which try to adjust the dataset to lessen the disparity between the sizes of the classes, are used to solve this problem. Two scenarios are offered in this regard: one that excludes instances from the majority class, known as under-sampling, and another that creates examples for the minority class, known as over-sampling [29]. Because random under-sampling has been shown to provide poor results in comparable studies, over-sampling strategies are frequently favored including Synthetic Minority Over-sampling Technique (SMOTE) which is commonly used in data balancing approaches [30].

SMOTE is an oversampling technique that uses synthetic cases to represent minority classes. The approach inserts synthetic samples along the line between the current instance and some of its k nearest neighbors from the same class for each minority class sample. Depending on how much oversampling is required, the algorithm selects some of the k nearest neighbors at random and creates pairs of vectors to produce fake samples. The new instances result in decision areas that are bigger and denser. This allows classifiers to learn more from the smaller classes in those decision zones rather than the much larger classes that surround them. SMOTE, according to Wang et al, outperforms other over sampling algorithms [31].

### D) Performance metrics

Measuring how well a classification model predicts the intended outcome is critical when developing and optimizing it. However, the accuracy metric by itself is never sufficient, as it might still produce false results. As a result, there are additional performance evaluations to aid in the interpretation of the model [32]:

*True positive (TP)*: The observation is truly positive and is predicted as positive.
*False Negative (FN)*: The observation is truly positive and is predicted to be negative.
*True Negative (TN)*: The observation is truly negative and is predicted as negative.
*False Positive (FP)*: The observation is truly negative while it is predicted as positive.

In practice, the cost of false negatives differs from the cost of false positives, depending on the circumstances. It goes without saying that we should not only calculate accuracy, but also evaluate our model using other metrics like recall and precision.

#### F1 score

At the expense of the other statistical measure, we aim to optimize either recall or precision. However, in circumstances where we wish to discover the best balance of precision and recall, we can use the F1 score to combine the two criteria [33, 34].

#### Coherence score

Subject coherence is a metric that assesses the degree of semantic similarity between high-scoring terms in a single topic. These metrics help differentiate between semantically interpretable subjects and topics that are statistical inference artefacts [35].

Topic coherence score is usually used to determine the appropriate number of topics to be extracted using LDA to determine how effectively the topics are extracted:

$$\mathrm{CoherenceScore}=\sum_{i<j}SCORE_{UMass}(w_{i},w_{j})$$ (1)

where *w*_*i*_ and *w*_*j*_ are the top words of the topic and Intrinsic UMass measure is

$$SCORE_{UMass}(w_{i},w_{j})=log\frac{D(w_{i},w_{j})+1}{D(w_{i})}$$ (2)

The coherence score is for assessing the quality of the learned topics.

This makes sense as a metric of subject coherence, because if two terms in a topic truly belong together, they should appear together frequently. The denominator simply accounts for the frequency of the words you’re evaluating in the documents, ensuring that terms like "the" don’t receive an unnaturally high score [36].

The topic coherence scores, *CS*(*t*) for t = 1…, K, can be used to determine the optimal number K* of topics by finding $arg\;\underset{K}{max}\frac{1}{K}\sum_{t=1}^{K}\;CS(t)$. That can be achieved by taking the average topic coherence score for various settings of K and seeing which gives the highest average coherence.

### E) Topic classification

It is a generic machine learning approach that aims to improve predictive performance by combining predictions from various models. Although there appears to be no limit to how many ensembles might be created for the predictive modelling task. In most cases, ensemble approaches yield more accurate results than a single model. Most ensemble approaches create homogeneous base learners using a single base learning algorithm. On the other hand, some ensemble approaches utilize a combination of heterogeneous learners, employing different base learning algorithms. To achieve higher accuracy compared to individual predictors, ensemble techniques require accurate and diversified base learners. It is crucial for ensemble techniques to incorporate base learners that are as precise and diverse as possible [37].

In this research several commonly used machine learning techniques were used to classify the topics present in the COP9 tweets as shown in Fig 1B. Those techniques are Logistic Regression, Naïve Bayes, SVM, XGboost, Random Forest, and the Extra Trees.

Logistic Regression is a type of regression analysis used to predict the probability of a binary outcome, by fitting data to a logistic curve. It is simple and efficient but assumes that the relationship between the features and the target variable is linear. Naïve Bayes is a probabilistic algorithm that uses Bayes’ theorem to predict the probability of a given sample belonging to a particular class, based on its features. It assumes that the features are independent of each other, which is not always the case in real-world data. SVM is a popular algorithm that separates data into classes by finding the best hyperplane that maximizes the margin between them. It is effective for high-dimensional data and can handle non-linear decision boundaries but may overfit when the data is noisy or overlapping. XGBoost is a gradient boosting algorithm that combines the predictions of many weak models to improve accuracy. It is highly efficient and can handle large datasets with complex structures but may suffer from overfitting if not properly tuned. Random Forest is a decision tree ensemble method that creates multiple decision trees using bootstrap samples of the data and random feature subsets. It is highly accurate and can handle noisy data and high-dimensional features. While Extra Trees is a variation of random forests that creates multiple decision trees using random feature subsets and splitting points. It is less prone to overfitting than random forests but may have higher bias due to the randomness introduced in the model [38].

Knowing the labels of each tweet that represent their dominant topics derived with an unsupervised machine learning technique, namely, LDA can allow the supervised machine learning techniques classifiers to predict those labels after a training stage.

### F) Sentiment analysis

RNN (Recurrent Neural Network) solves memory problems by providing a feedback mechanism that compares the current output to the prior output and acts as a form of memory. Because the prior outputs acquired during training leave a trace, the model can easily predict future tokens (outputs) using the previous ones [39].

**Fig 2:**
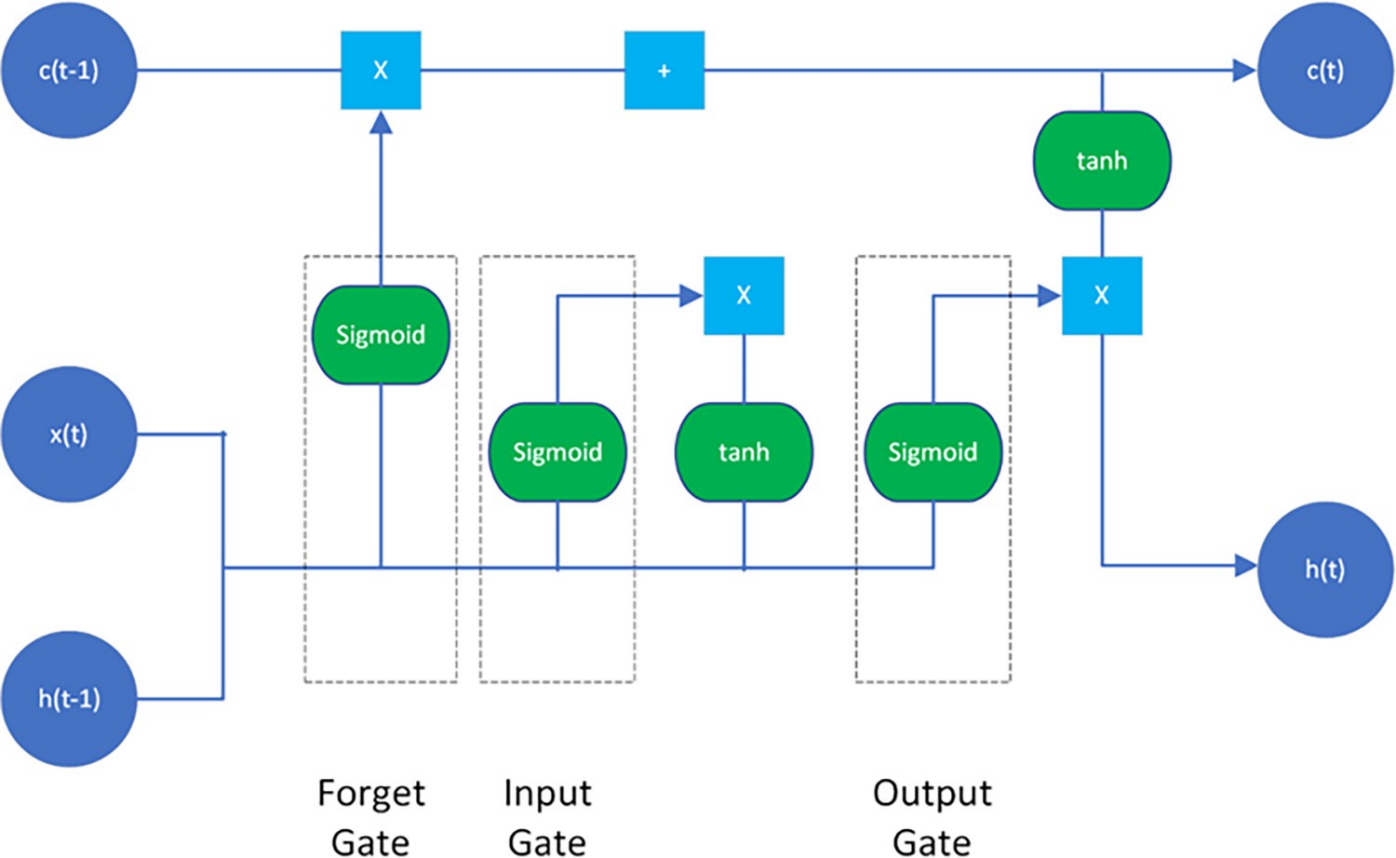
The LSTM architecture.

Every word in a sentence or phrase has significance only if it is connected to the word before it and the word after it. LSTM (Long Short-Term Memory) expands RNN by including both short- and long-term memory components to examine and remember sequential material more quickly [40]. As a result, it’s ideal for things like Machine Translation, Speech Recognition, and time-series analysis as shown in Fig 2.

Every unit of the LSTM network is known as a “cell”. The following equations describe each cell:

- Forget gate (what info to throw away from previous steps):

$$f_{t}=\sigma (W_{f}[h_{t-1},x_{t}])$$ (3)

- Input gate (what new info will be stored in the memory cell):

$$i_{t}=\sigma (W_{i}[h_{t-1},x_{t}])$$ (4)

- New memory cell candidate values:

$${\tilde{C}}_{t}=tanh(W_{C}[h_{t-1},x_{t}])$$ (5)

- Update memory cell (using input and output gates):

$$C_{t}=f_{t}*C_{t-1}+i_{t}*{\tilde{C}}_{t}$$ (6)

- Output (decide what’s the output filtered by the memory cell):

$$o_{t}=\sigma (W_{o}[h_{t-1},x_{t}])$$ (7)

$$h_{t}=o_{t}*tanh(C_{t})$$ (8)

Where:

- *x*_*t*_:  token at timestamp t,
- *h*_*t*−1_:  previous hidden state,
- *C*_*t*−1_ :  previous cell state,
- *h*_*t*_ :   updated hidden state,
- *C*_*t*_:   current cell state

LSTM utilizes a distinct concept for controlling the memory retention process. The gates in LSTM, also known as the gating mechanism, store the memory components in analog format and convert them to a probabilistic score by performing point-wise multiplication with the sigmoid activation function, which stores it in the range of 0–1. The flow of information in and out of LSTM cells is controlled by gates [41].

Because the only inputs it has seen are from the past, the unidirectional LSTM only retains information from the past. Using bidirectional will run the inputs in two directions, one from the past to the future and the other from the future to the past, providing the ability to store information from both the past and the future at any point in time [42]. Therefore, Bi-LSTMs produce excellent results since they are better at understanding context.

Three correlation metrics were applied to assess the correlation between retweets and sentiment scores (just for the tweets having the dominant topic labelled as “harm reduction”) to predict possible solutions discussed in Tweets as shown in Table 1.

**Table 1:** Applied correlation metric.

Correlation Metric	Definition
**Pearson**	Canonical parametric measure of correlation between variables, assuming linear relationship [43].
**Kendall Rank**	Non-parametric measure of correlation between ranks of variables [44].
**Spearman Rank**	Non-parametric measure of correlation between ranks of variables, assuming monotonic relationship [45, 46].

### G) Toxicity analysis

In the domain of sentiment analysis, various approaches have been developed to assess the toxicity and offensiveness of text samples. One notable tool is Google’s Perspective API, which utilizes deep learning models to classify text and predict whether a message is toxic or not. This API provides a simple and accessible way for developers and publishers to analyze social media conversations and provide real-time feedback to commenters [47]. It’s worth noting that the Perspective API has been updated, and as of February 2020, its performance in terms of toxicity classification is significantly improved, approaching the results of other advanced models such as the Bi-GRU with attention mechanism model. This update enhances the effectiveness of Perspective in identifying and addressing toxic content in real-time social media interactions [48].

By assessing a statement across several emotional concepts, toxicity analysis determines the potential effect a comment may have on a discussion. A probability rating of the toxicity score reflects the likelihood that a reader will interpret the request’s remark as possessing the specified property. The ratings offered for each attribute reflect a likelihood, with a value ranging from 0 to 1. The chance that a reader will consider the comment to possess the provided attribute increases with a higher score. For the characteristic of toxicity, a probability score of 0.7 can mean that 7 out of 10 individuals would consider the statement to be toxic [49].

Toxicity in the social realm is defined as the propagation of unneeded negativity or hatred that has a detrimental impact on the mental well-being, emotional state, and overall experience of those who are exposed to it [50]. The toxicity was measured in the filtered “harm reduction” tweet data to indicate the level of neutrality and reasonability, and whether the language used in the online discussions was harmful, offensive, or potentially triggering to individuals.

## Results and discussion

### A) Topic modelling

**Fig 3:**
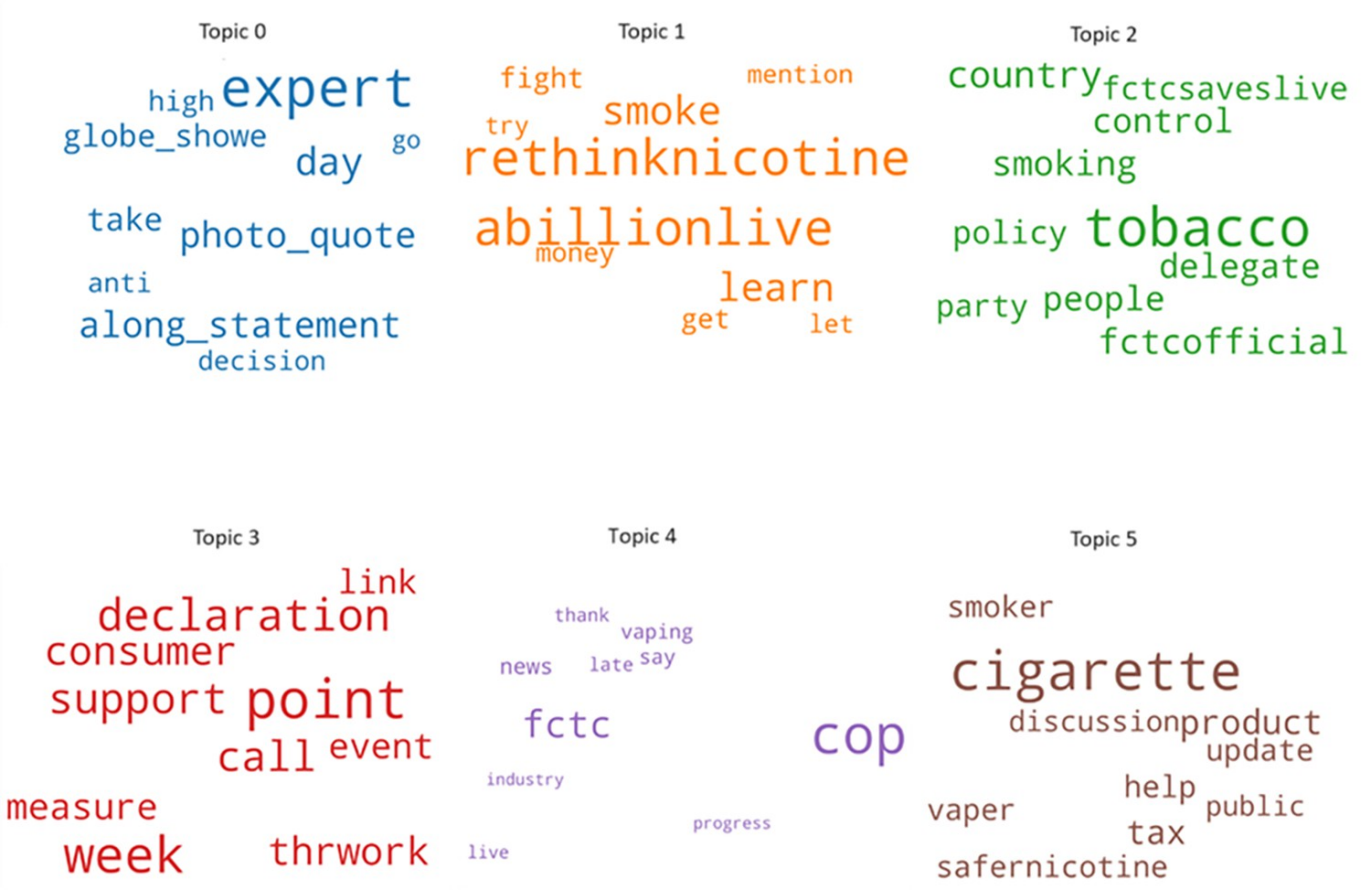
Word clouds for the identified six topics.

**Fig 4:**
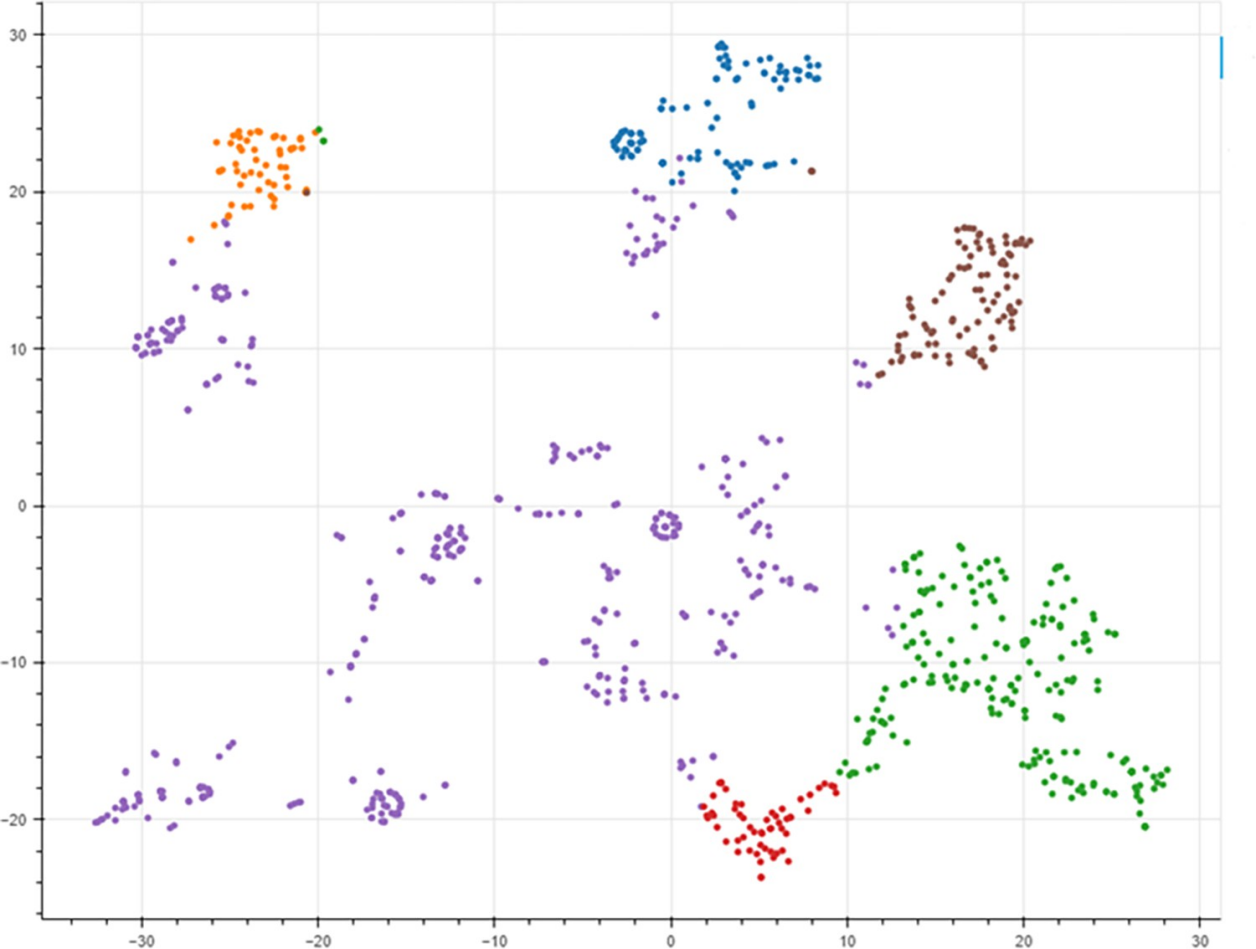
The t-SNE visualization for the six topics present in the COP9 dataset.

Six different topics were identified in the COP9 twitter dataset, corresponding to a maximum coherence score in the tweets’ corpus. Their corresponding word clouds are shown in Fig 3, with the t-SNE visualization presented in Fig 4 illustrating the spatial distribution of the six distinct topics within the COP9 dataset, effectively capturing their positioning across a two-dimensional space. This latter visualization offers valuable insights into the arrangement and relationships among the topics. The six topics, with their associated top 10 words and suggested labels, are shown in Table 2.

- *Expert Opinion and Decision-*Making: This topic cluster involves discussions related to expert opinions and decision-making. Conversations within this cluster likely revolve around decisions made by experts, possibly in the context of tobacco control policies.
- *Anti-Smoking Initiatives and Advocacy*: This cluster focuses on anti-smoking initiatives and advocacy efforts. Conversations here are likely centered on campaigns against smoking and efforts to raise awareness about its harmful effects.
- *Tobacco Control Policies and Delegates*: This topic pertains to discussions about tobacco control policies and the involvement of various stakeholders, including countries and delegates. Conversations within this cluster likely revolve around policies related to tobacco control.
- *Consumer Support and Measures*: In this cluster, discussions are related to consumer support and measures taken in some context. Conversations here may be about supporting consumers and implementing measures, potentially within the tobacco control domain.
- *Conference (COP) and Industry*: This topic is associated with discussions regarding conferences, particularly the Conference of the Parties (COP), and the tobacco industry. Conversations within this cluster may revolve around events related to COP and developments within the tobacco industry.
- *Harm Reduction*: The "Harm Reduction" cluster predominantly delves into discussions related to harm reduction, with a particular emphasis on tobacco and smoking. Key terms within this cluster include "cigarette," "product," "tax," "help," "smoker," and "safer nicotine." These terms are commonly linked to conversations concerning strategies aimed at reducing harm within the tobacco and nicotine product domain. Within this cluster, conversations frequently revolve around debates regarding safer nicotine alternatives, potential tax policies, and initiatives designed to aid smokers in minimizing the harm associated with tobacco use.

**Table 2:** The top 10 words for each topic cluster.

Topic/Top Words	1	2	3	4	5	6	7	8	9	10	Label
**0**	expert	day	along_statement	photo_quote	take	globe_show	high	anti	decision	go	"Expert Opinion and Decision-Making"
**1**	rethinknicotine	abillionlive	smoke	learn	fight	get	money	let	mention	try	"Anti-Smoking Initiatives and Advocacy"
**2**	tobacco	country	fctcofficial	smoking	people	delegate	control	policy	fctcsaveslive	party	"Tobacco Control Policies and Delegates"
**3**	point	week	declaration	support	call	consumer	thrwork	measure	event	link	"Consumer Support and Measures"
**4**	cop	fctc	news	say	vaping	late	thank	live	progress	industry	“Conference (COP) and Industry”
**5**	cigarette	product	tax	help	smoker	update	vaper	safernicotine	discussion	public	“Harm Reduction”

In this research we have focused on harm reduction as a primary concern of policy makers for tobacco control. Therefore, the tweets associated with Topic 5 were filtered in for further processing.

### B) Topic classification

The number of tweets belonging to each topic varies significantly resulting in an imbalanced dataset as shown in Table 3. Therefore, a SMOTE technique was applied to balance the dataset to achieve better results when applying the classification models [51].

**Table 3:** The number of tweets per topic.

Topic	Tweets
**0**	793
**1**	512
**2**	1456
**3**	452
**4**	3196
**5**	967

The following step was to apply commonly used machine learning techniques to classify the six topics associated with the COP9 tweets which can be a highly effective way to analyze and categorize large amounts of data. These techniques included supervised learning algorithms namely, decision trees, support vector machines, and logistic regression. By training these algorithms on a set of labeled data, they can learn to recognize patterns and make predictions about the category of new, unlabeled data. Those techniques were designed and implemented using a data split of 70:30. Additionally, 10-fold cross-validation was performed to evaluate the models.

The highest accuracy achieved for testing was 91.87% and an F1-score of 0.9256 using Random Forest which reflects reasonable performance for classifying the six topics in the dataset. A comparison of the applied machine learning models’ performance metrics was summarized in Table 4.

**Table 4:** The macro average performance of topic prediction for the applied machine learning classifiers.

Model	Accuracy	Precision	Sensitivity	Specificity	F1-Score
**Logistic Regression**	0.8873	0.8949	0.8844	0.8896	0.8896
**Naive Bayes**	0.8730	0.9037	0.8522	0.8967	0.8772
**SVM**	0.8523	0.8588	0.8689	0.8330	0.8638
**XGboost**	0.9023	0.9129	0.9090	0.8942	0.9109
**Extra Trees**	0.8943	0.8961	0.8979	0.8906	0.8970
**Random Forest**	0.9187	0.9166	0.9347	0.8998	0.9256

### C) Tweets’ sentiments

**Fig 5:**
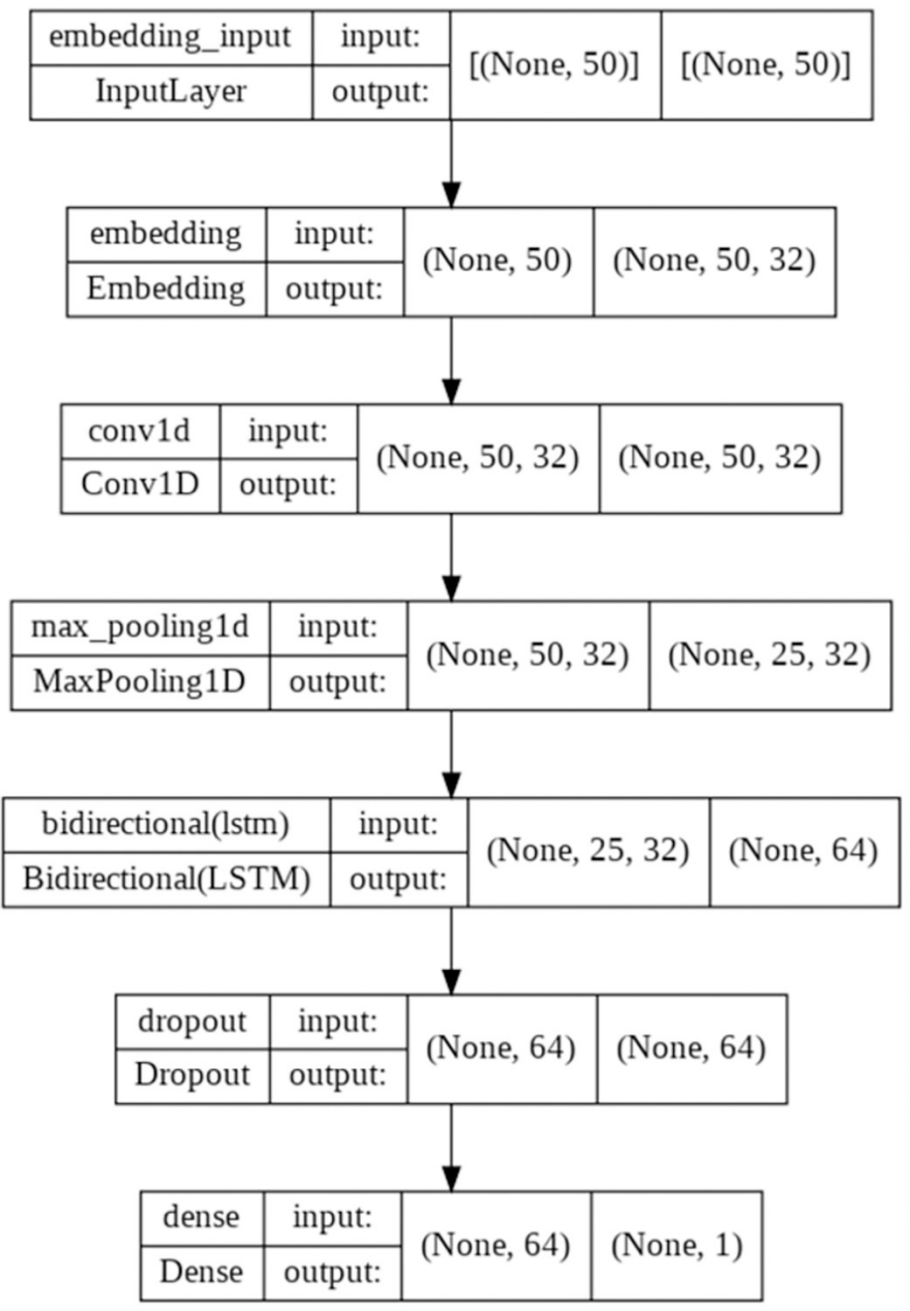
The Bi-LSTM architecture used in the sentiment analysis step.

The Bi-LSTM architecture designed for sentiment analysis is illustrated in Fig 5. In this research, 60% of the dataset was designated for training, 20% for validation, and the remaining 20% for testing. The deep learning model for sentiment analysis was trained using the IMDB dataset.

For a comprehensive understanding of the model’s configuration, the following hyperparameters were employed: a batch size of 16 was chosen to efficiently process the data, the model was trained over 50 epochs to optimize its performance, a dropout rate of 0.2 was incorporated to prevent overfitting, and the sigmoid activation function was used for most nodes in the model, while the activation function for the output layer was the tanh function to accommodate the desired sentiment prediction ranges. The learning rate was set to 0.00001 to control the rate of parameter updates. The achieved testing performance of the designed Bi-LSTM to predict the sentiments of the IMDB reviews dataset resulted in an F1-Score of 0.8908 as shown in Table 5.

**Table 5:** The model accuracy for the Bi-LSTM classifier.

Sentiment	Precision	Recall	F1-score	Support
**0**	0.8894	0.8769	0.8831	5035
**1**	0.8950	0.9020	0.8985	4965
**Accuracy**			0.8930	10000
**Macro Avg**	0.8922	0.8895	0.8908	10000
**Weighted Avg**	0.8922	0.8894	0.8908	10000

In the process of refining the Bi-LSTM sentiment analysis model for COP9-related tweets, the fine-tuning stage assumes a pivotal role. This phase encompasses a series of steps aimed at tailoring the model to precisely analyze the sentiment within these tweets. To initiate this process the text data is meticulously preprocessed, a crucial step involving the removal of extraneous elements like stop words and punctuation. Subsequently, the annotated dataset is partitioned into training, validation, and test sets, maintaining an 80:10:10 ratio.

Leveraging insights garnered from the trained Bi-LSTM model originally developed for the IMDB dataset, we incorporate it into our architecture. In this fine-tuning process, all layers of the trained model are frozen except for the output layer. This approach allows valuable knowledge encoded in the trained layers to be maintained while adapting the model specifically to the COP9-related sentiment analysis task. Moreover, the same hyperparameters utilized for the trained Bi-LSTM model are employed, ensuring consistency in the fine-tuning process. This approach facilitates the enhancement of the sentiment analysis model’s accuracy, making it adept at precisely capturing sentiment nuances within the COP9-related tweets.

Table 6 presents the performance metrics for both the trained Bi-LSTM based on IMDB and the fine-tuned Bi-LSTM based on annotated COP9, including accuracy, precision, recall, and F1 score for each sentiment class. The fine-tuned model demonstrates improved sentiment analysis accuracy compared to the base model. Therefore, the resulting fine-tuned Bi-LSTM model significantly enhances the accuracy of sentiment analysis for COP9-related tweets, contributing to a more insightful understanding of public sentiment associated with tobacco domain.

**Table 6:** Performance of both trained Bi-LSTM based on IMDB and fine-tuned Bi-LSTM based on annotated COP9.

Model	Accuracy	Precision			Recall			F1 Score		
		Negative	Neutral	Positive	Negative	Neutral	Positive	Negative	Neutral	Positive
**Bi-LSTM**	0.7920	0.8410	0.6710	0.8071	0.8124	0.7102	0.7815	0.8265	0.6900	0.79409
**Fine-tuned Bi-LSTM**	0.8311	0.8641	0.7421	0.8312	0.8311	0.7613	0.8212	0.8473	0.7516	0.8262

**Fig 6:**
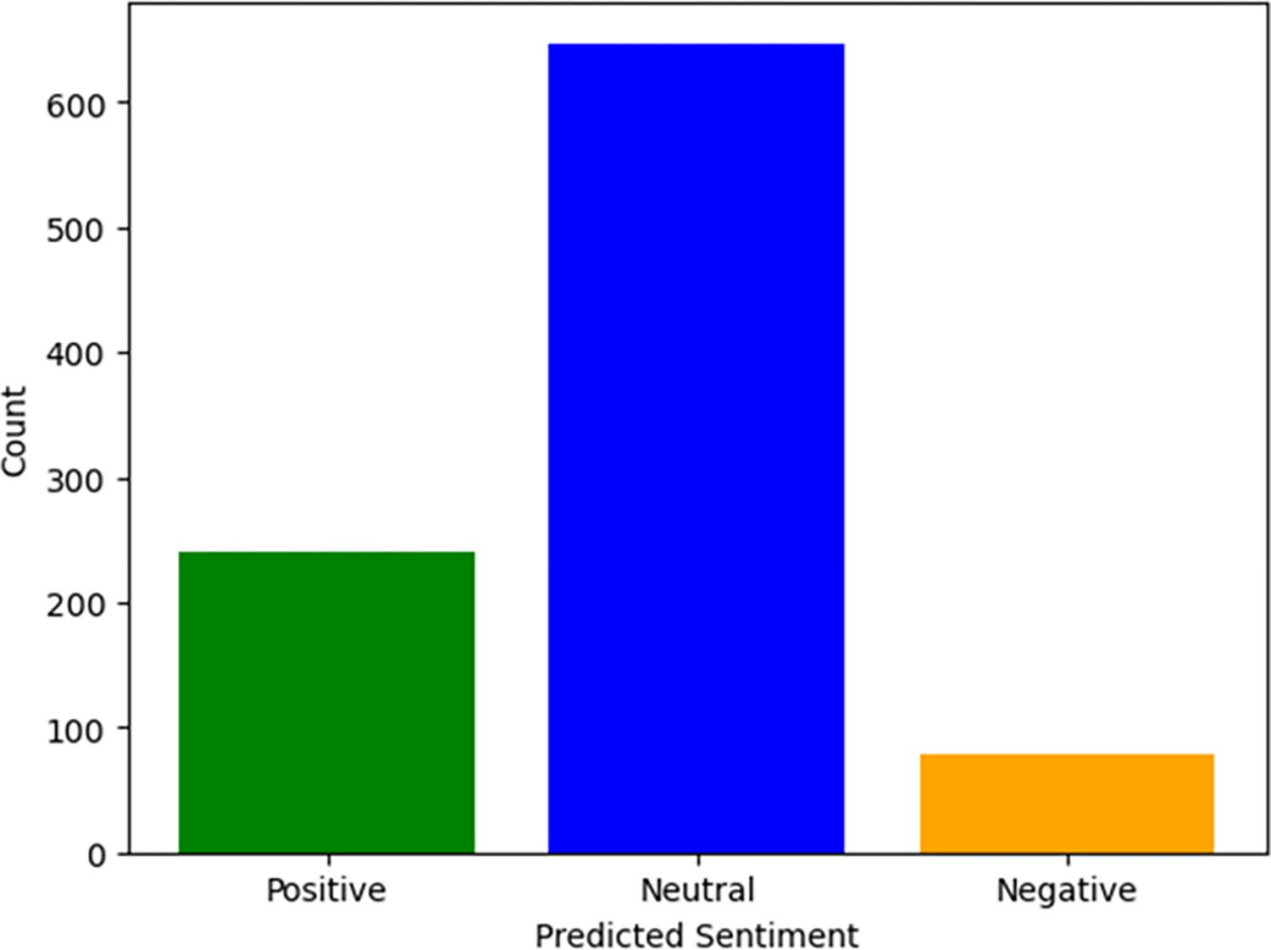
Sentiment scores using the fine-tuned Bi-LSTM.

Fig 6 illustrates the application of sentiment analysis using the fine-tuned Bi-LSTM on a selected group of tweets focused on topic 5, identified as "harm reduction." These tweets were filtered from the larger dataset to exclude irrelevant content. Sentiment scores were assigned as Negative when the score was less than or equal to -0.5, Positive when the score was greater than or equal to 0.5, and Neutral for sentiment scores falling in-between.

According to the analysis, most of the tweets in the COP9 dataset labelled as “harm reduction” exhibit a neutral sentiment. This indicates that the complexity of the discussions surrounding the topic of “harm reduction” during COP9 in these tweets cannot be characterized as either strongly supportive or strongly critical. There are many ways this could be interpreted. This may simply reflect a sharing of information and/or opinion in a neutral manner using somewhat muted language. Alternatively, the neutral tone observed may reflect a strategic effort to advance a “harm reduction” narrative, or vice-versa, but without explicit advocacy, thus appearing neutral. Ultimately, sentiment analysis such as this provides an initial snapshot of the prevailing tone in the dataset, which then can prompt further in-depth analysis if required. Furthermore, tweets from a limited period around COP9 alone offer only one restricted perspective on discussions around “harm reduction” and cannot be used to comprehensively characterize the nature and differing agendas of the wider “harm reduction” debate.

**Fig 7:**
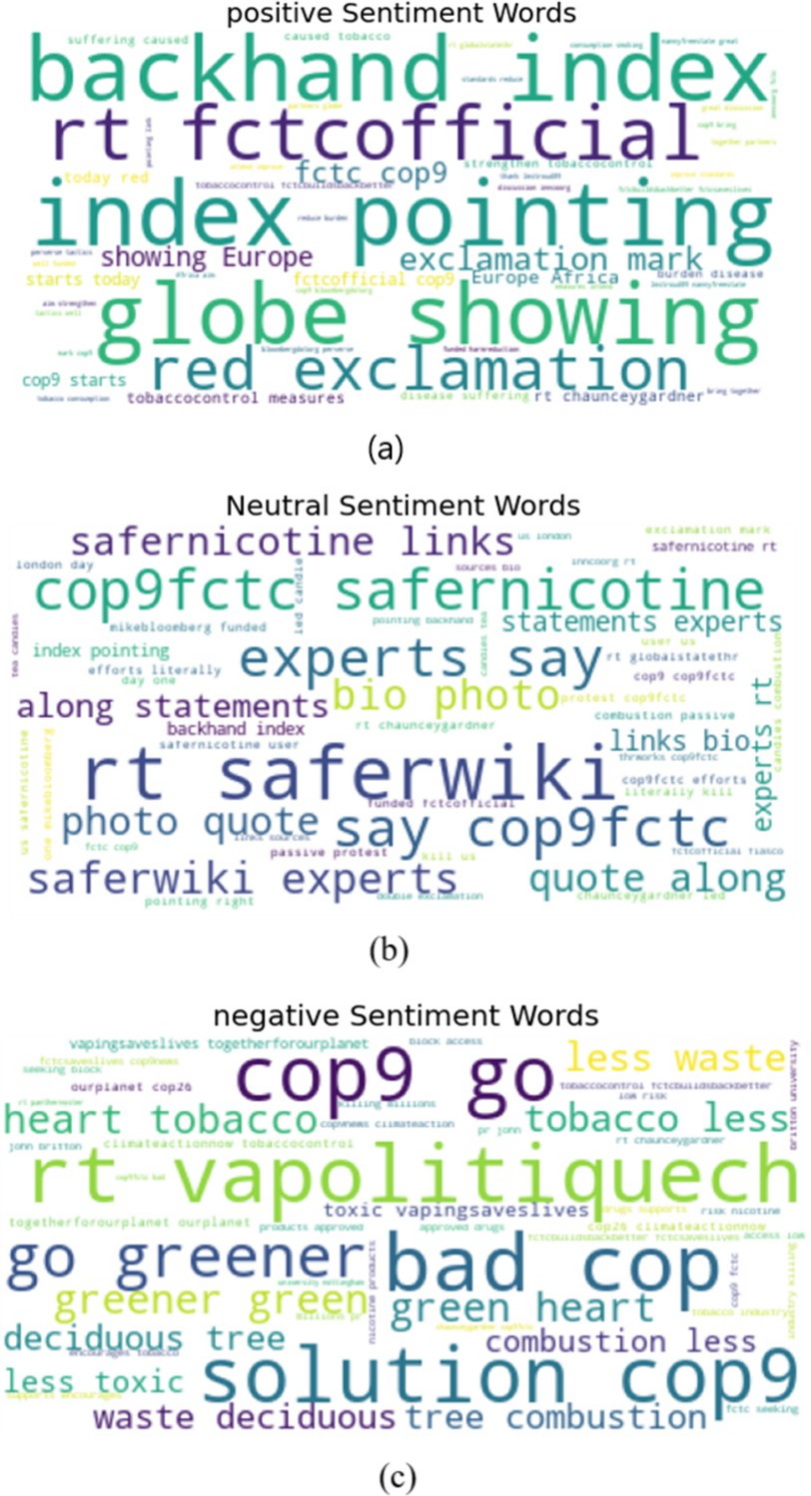
The word cloud (a) positive, (b) neutral, and (c) negative tweets in the COP 9 “harm reduction” tweets.

The word cloud for the three sentiments found in the “harm reduction” topic, after being assigned a sentiment score using the fine-tuned Bi-LSTM, were then identified, and shown in Fig 7 reflecting important words present in the tweets with different sentiments.

Identifying prevalent sentiment trends enables one to stay informed about how public opinion is evolving over time and moreover help policymakers make informed decisions and tailor their approaches, particularly around important topics of interest such as “harm reduction”.

### D) Retweets count and sentiment score correlation

The retweets within the dominant topic “harm reduction” were categorized based on their retweet count, and the corresponding sentiment absolute score values were averaged. Table 7 presents the relationship between the retweet count and the average of absolute sentiment scores.

**Table 7:** Retweets vs average sentiment score.

Retweets	Avg Sentiment Score
4	0.359497
5	0.446233
6	0.593756
7	0.673377
8	0.743518
9	0.727569
10	0.6978501
11	0.741540
13	0.758641
14	0.783395
17	0.750989
19	0.570859
20	0.810161

After applying Pearson, Kendall, and Spearman correlation metrics, the results were significant and the association between the two variables would be considered statistically significant as shown in Table 8. Therefore, we can conclude that the higher the tweet’s absolute sentiment score, the higher the probability that it is likely to be retweeted.

**Table 8:** Pearson, Kendall rank, and Spearman correlation.

Correlation	Correlation Coefficient	p-value
**Pearson**	0.577837	0.038611
**Kendall rank**	0.615385	0.002676
**Spearman**	0.697802	0.008001

### E) Toxicity analysis

In Table 9, we present the results of the toxicity analysis conducted on the filtered tweets with the dominant topic “harm reduction”. The average toxicity scores for the tweets were found to be relatively low, indicating that the language used in the online discussion was mostly non-toxic.

**Table 9:** Toxicity analysis for the tweets labelled as dominant topic “harm reduction”.

Analysis	Average for dataset	Highest value	Lowest value
**Toxicity**	0.08	0.39	0.00
** Severe Toxicity**	0.01	0.09	0.00
**Identity Attack**	0.03	0.18	0.01
**Insult**	0.07	0.52	0.00
**Profanity**	0.03	0.32	0.00
** Threat**	0.05	0.62	0.01

## Conclusions

The research conducted in this paper applied a three-stage analysis, namely topic modelling, topic prediction, and sentiment and toxicity analysis. An automated method was used based on LDA techniques to extract suitable features from the tweets in the dataset to allow filtering of the tweets by dominant topic. The LDA was chosen due to the nature of the tweets that might have overlapping topics. The machine learning classification stage was used to classify labelled tweets into their associated topics and filter in those tweets with a dominant topic labelled as “harm reduction”. The last stage was the sentiment analysis which was performed using Bi-LSTM. The methodology then scored the tweet sentiment to find the correlation between the number of retweets and the absolute value of average tweet sentiment, resulting in a significant correlation between the number of retweets and the sentiment of those tweets. This methodology can facilitate analyzing large tweets datasets and highlighting key topics within them.

A high-accuracy topic classifier plays a crucial role in the context of research on tobacco control by filtering and identifying relevant topics within narratives associated with events like COP in real time. This capability allows focus to be given to discussions that are most pertinent to the research domain and enabling the impact of these identified topics to be assessed. The real-time operation of the classifier is valuable for monitoring ongoing discussions and tracking how topics evolve over time, providing insights into event dynamics and changing public sentiment or interest. In essence, the classifier enhances the ability to analyze and understand COP-related discussions, offering valuable insights into trending topics and their significance.

Moreover, a rapid response to the moving social media landscape surrounding events such as COP is imperative to inform policy makers with accurate information on public perceptions of policy issues. Analyzing social media data can also assist with highlighting online echo chambers attempting to stall or change the direction of regulation. A further step was taken in this methodology to analyze toxicity in the tweet language, which gives a better insight into the content of the online conversations. The toxicity analysis performed in this case showed that the tweet content was inoffensive as the average toxicity measures were relatively low, although several tweets were found to contain a level of toxic language which is expected in unfiltered online conversation.

The proposed paradigm is an attempt to make social media analysis easier by implementing a paradigm that can identify key topic areas and analyze language for specific topics of importance, such as “harm reduction”. The ability to focus analysis on certain topics will be useful for policy makers to quickly identify public understanding and opinion and how that opinion is formed.
